# Synthesis and NMR Spectral Studies of the 7-C_60_-Adduct of *N*,*N*-(Tetrachlorophthaloyl) Dehydroabietylamine

**DOI:** 10.3390/molecules17044209

**Published:** 2012-04-05

**Authors:** Zhi Zhou, Zhongxiang Lin

**Affiliations:** 1 College of Chemical Engineering, Nanjing Forestry University, Nanjing 210037, China; 2 College of Life and Environmental Sciences, Kaili University, Kaili, Guizhou 556011, China

**Keywords:** 7-C_60_-adduct of *N*,*N*-(tetrachlorophthaloyl)dehydroabietylamine, ^1^H-NMR, ^13^C-NMR, 2D NMR, *C_1_* symmetric structure

## Abstract

The 7-C_60_-adduct of *N*,*N-*(tetrachlorophthaloyl)dehydroabietylamine was synthesized for the first time and characterized by IR, UV-vis, mass and NMR spectral studies. The ^1^H-NMR and ^13^C-NMR resonance signals of the new compound are unambiguously assigned by using homo- and heteronuclear 2D NMR spectroscopic techniques such as COSY, ROESY, HSQC and HMBC. The *C_1_* symmetric structure with 6,6-junction of compound was determined.

## 1. Introduction

Dehydroabietylamine, which possesses an aromatic diterpene structure with three rings and a reactive amino group and is the main component of disproportionated rosin amine, can be easily isolated from the latter. Dehydroabietylamine and its derivatives have attracted considerable interest due to their wide range of uses such as chiral resolution agents [1,2], antibacterial substances [3,4] and chiral surfactants [5,6].

**Figure 1:**
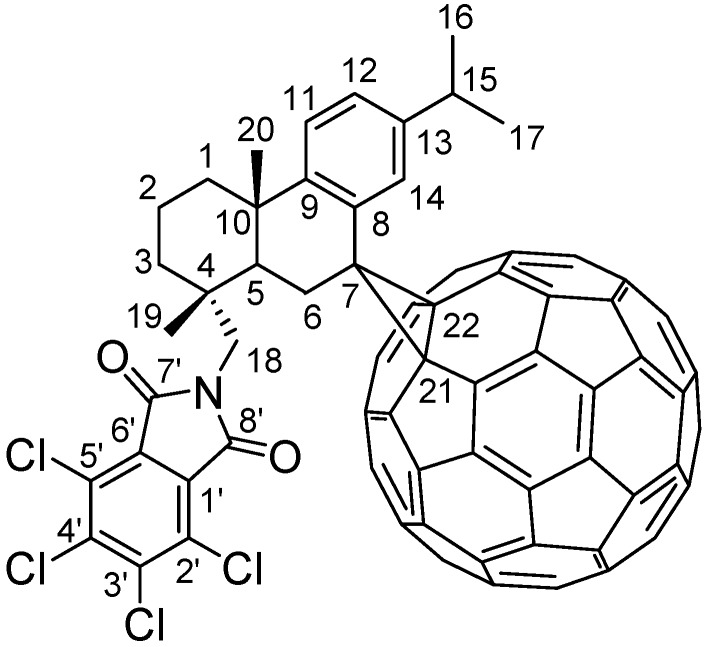
Chemical structure of 7-C_60_-adduct of *N,N*-(tetrachlorophthaloyl)dehydroabietylamine (**5**).

Since the discovery of C_60_, its peculiar cage structure has attracted great attention. Cycloaddition reactions of C_60_, especially 1,3-dipolar addition reactions, have been the subject of a large variety of studies and shown to be a useful methods for the synthesis of functionalized fullerene derivatives [7,8,9,10,11]. These results prompted us to study new cycloadducts of dehydroabietylamine with C_60_ as a part of an on-going program for the development of new rosin amine derivatives with potential biological or material properties. So far, the majority of the studies on the chemical transformations of dehydroabietylamine have focused on the amine group and benzene ring, but the chemical transformation in other skeleton has seldom been reported. In this paper, we report the synthesis for the first time of the 7-C60-adduct of *N,N*-(tetrachlorophthaloyl)dehydroabietylamine (**5**, Figure 1) and describe the structure determination of the new compound, along with its detailed ^1^H- and ^13^C-NMR assignments.

## 2. Results and Discussion

### 2.1. Synthesis Procedures

**Scheme 1:**
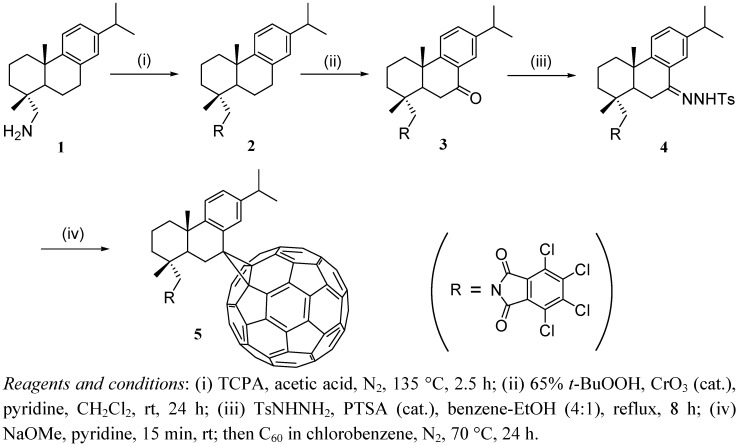
Preparation of compound **5**.

The general procedure for the synthesis of compound **5** is shown in Scheme 1. Dehydroabietylamine (**1**) was prepared as described in the literature [12]. Dehydroabietylamine reacted with tetrachlorophthalic anhydride (TCPA, an amino protecting group) to give compound **2**, which then was transformed into **3** by C-7 benzylic oxidation. Subsequently, the reaction of **3** with *p*-tosylhydrazide yielded *p*-tosylhydrazone **4**. Compound **5** was prepared according to the reported method [13]. According to the literature [13], heating a solution of the *p*-tosylhydrazone with C_60_ at 70 °C afford the [5,6]-open isomer. However, in our experiment, no traces of this expected [5,6]-open isomer were found and the only isolated product was the [6,6]-closed isomer (methanofullerene), which is likely due to a rearrangement of the 5,6-open isomer into the thermodynamically more stable 6,6-closed isomer at 70 °C.

### 2.2. Analysis of Compound ***5***

#### 2.2.1. IR, UV-Vis and Mass Spectrum Analysis of Compound **5**

The IR spectrum of compound **5** showed bands indicating the presence of the different expected functional groups: Characteristic asymmetric and symmetric stretching bands of the imide carbonyl (C=O) at 1,776 and 1,721 cm^−^^1^ and at 526 cm^−^^1^ for the C_60_ skeleton. In the UV-vis spectrum, the characteristic absorptions for the methanofullerenes was also observed at 436.50, 697.00 nm [13]. Meanwhile, the structure of compound **5** as a monoadduct was supported by the matrix-assisted laser desorption/ionization time of flight mass spectrum (MALDI-TOF MS) which display the expected peak at *m/z* 1,271.4.

#### 2.2.2. ^1^H-NMR Spectrum Analysis of Compound **5**

The ^1^H-, ^13^C-NMR and 2D NMR spectra for compound **5** are summarized in Table 1.

**Table 1:** ^1^H- and ^13^C-NMR data, ^1^H-^1^H correlations in COSY, ROESY spectra and ^1^H-^13^C correlations in HSQC, HMBC spectra for compound **5**.

C	*δ*_C_ (ppm)	H	*δ*_H_ (ppm)	HSQC	HMBC	COSY	ROESY
1	39.48	1*β*	2.39 (br d, *J* = 11.9 Hz)	H-1*β*, 1*α*	H-20, 2, 3*α*	H-1*α*, 2	H-20, 11, 2, 1*α*
		1*α*	1.79–1.74 (m)			H-1*β*, 2	H-5, 11, 1*β*
2	18.18	2	1.85–1.79 (m, 2H)	H-2		H-3*β*, 3*α*, 1*β*, 1*α*	H-19, 3*β*, 3*α*, 1*β*
3	37.52	3*β*	1.58 (br d, *J* = 13.0 Hz)	H-3*β*, 3*α*	H-19, 18*α*, 18*β*	H-3*α*, 2	H-19, 3*α*, 2
		3*α*	1.53–1.47 (m)			H-3*β*, 2	H-5, 18*α*, 3*β*, 2
4	40.89	―	―	―	H-19, 5, 18*α*, 18*β*, 6*β*	―	―
5	48.67	5	1.97 (dd, *J* = 10.3, 9.2 Hz)	H-5	H-19, 20, 18*α*, 18*β*, 6*α*, 6*β*	H-6*β*, 6*α*	H-3*α*, 1*α*, 6*α*, 18*α*, 2
6	28.35	6*β*	3.65 (dd, *J* = 14.1, 10.5 Hz)	H-6*β*, 6*α*	C-6/H-5	H-5, 6*α*	H-19, 20, 6*α*
		6*α*	3.41 (dd, *J* = 14.1, 8.8 Hz)			H-5, 6*β*	H-5, 6*β*, 18*α*
7	44.61	―	―	―	H-6*α*, 6*β*, 14, 11	―	―
8	132.15	―	―	―	H-6*β*, 11,	―	―
9	150.35	―	―	―	H-14, 12, 20, 5	―	―
10	38.83	―	―	―	H-20, 5, 6*α*, 2, 11	―	―
11	123.40	11	7.43 (d, *J* = 8.1 Hz)	H-11	H-12	H-12	H-1*β*, 1*α*, 12
12	126.09	12	7.28 (dd, *J* = 8.1, 1.8 Hz)	H-12	H-15, 11, 14	H-11	H-15, 11, 16
13	145.43	―	―	―	H-16, 17, 15, 11	―	―
14	126.42	14	8.40 (d, *J* = 1.8 Hz)	H-14	H-12, 15	―	―
15	33.92	15	2.99 (septet, *J* = 6.7 Hz)	H-15	H-16, 17, 12, 14	H-16, 17	H-16, 17, 12
16	23.70	16	1.30 (d, *J* = 6.8 Hz, 3H)	H-16	H-15, 17,	H-15	H-15, 12
17	24.49	17	1.32 (d, *J* = 7.0 Hz, 3H)	H-17	H-15, 16	H-15	H-15
18	50.45	18*β*	3.88 (d, *J* = 13.6 Hz)	H-18*β*, 18*α*	H-19, 5	H-18*α*	H-18*α*, 19
		18*α*	3.59 (d, *J* = 13.6 Hz)			H-18*β*	H-18*β*, 5, 3*α*, 6*α*
19	18.89	19	1.31 (s, 3H)	H-19	H-5, 18*α*, 18*β*	―	H-20, 6*β*, 18*β*, 3*β*, 2
20	22.50	20	1.86 (s, 3H)	H-20	H-1*α*, 5	―	H-19, 6*β*, 1*β*
21,22	84.80	―	―	―	H-6*α*, 6*β*	―	―
	77.66	―	―	―	H-6*α*	―	―
1',6'	127.52	―	―	―	―	―	―
2',5'	129.76	―	―	―	―	―	―
3',4'	140.35	―	―	―	―	―	―
7',8'	164.51	―	―	―	H-18*α*, 18*β*	―	―
C_60_	150.74–137.82	―	―	―	―	―	―

**Figure 2:**
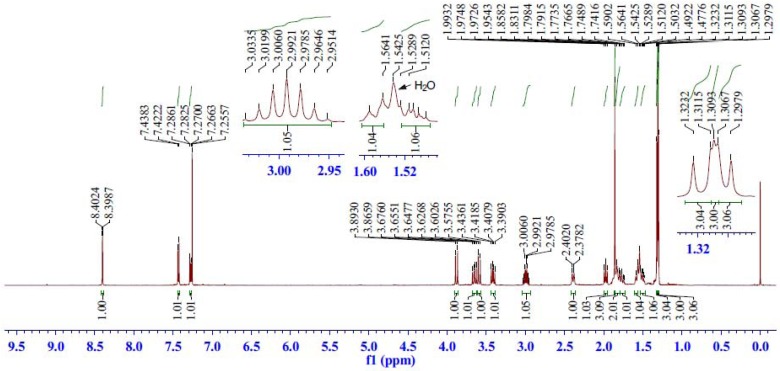
^1^H-NMR spectrum of compound **5**.

First, the assignment of some protons is easily accomplished by analysis of the ^1^H-NMR chemical shifts, signal multiplicity and coupling constants. The signals of the aromatic protons (7.26–8.40 ppm) can be readily identified by their chemical environment (Figure 2). 

The H-11 signal appeared as a doublet with coupling constants *J*_11,12_ = 8.1 Hz at 7.43 ppm, while the H-14 signal appeared at 8.40 ppm as a doublet with a lower coupling constant values *J*_14,12_ = 1.8 Hz because of the long distance between H-14 and H-12. H-12 signal appeared as a double doublet with *J*_12,11_ = 8.1 and *J*_12,14_ = 1.8 Hz at 7.28 ppm. The septet at 2.99 ppm (*J* = 6.7 Hz) was assigned to H-15 proton. In the higher frequency region there are three double doublets and two doublets at *δ*_H_ 3.65 (dd, *J* = 14.1, 10.5 Hz), 3.41 (dd, *J = *14.1, 8.8 Hz) 1.97 (dd, *J* = 10.3, 9.2 Hz), 3.88 (d, *J* = 13.6 Hz) and 3.59 ppm (d, *J* = 13.6 Hz). Of the five signals, *δ*_H_ 3.88 and 3.59 ppm doublets were assigned to H-18 (H-18*β* and H-18*α*) on the basis of the correlations of *δ*_C_ 50.45 ppm/H-18*β*, 18*α* in the HSQC spectrum, while *δ*_H_ 3.65 and 3.41 ppm double doublets were assigned to the H-6 (H-6*β* and H-6*α*) on the basis of the correlations of *δ*_C_ 28.35 ppm/H-6*β*, 6*α* in the HSQC spectrum. The remaining double doublet at *δ*_H_ 1.97 ppm was assigned to H-5. To distinguish the non-equivalent H-18 and H-6 protons, we found that *δ*_H_ 3.59 and 3.41 ppm showed ROE correlations with H-5, but *δ*_H_ 3.88 and 3.65 ppm showed no ROE correlations with H-5 in the ROESY spectrum. Therefore, we assigned H-18*α* at *δ *3.59, H-6*α* at *δ *3.41, H-18*β* at *δ *3.88 and H-6*β* at *δ *3.65 ppm.

The signals of four-methyl group protons appeared as two singlets and two doublets. The two singlets, one at *δ*_H_ 1.86 ppm was assigned to H-20 methyl protons, and the other at *δ*_H_ 1.31 ppm was assigned to H-19 methyl protons based on the correlations of *δ*_H_ 1.86/H-19, 6*β* and *δ*_H_ 1.31/H-20, 6*β*, 18*β* in the ROESY spectrum. The doublet at *δ*_H_ 1.30 ppm was readily assigned to H-16 methyl protons in the isopropyl moiety due to the *δ*_H_ 1.30/H-15 correlation in the COSY spectrum and *δ*_H_ 1.30/H-15, 12 correlations in the ROESY spectrum. The remaining doublet at *δ*_H_ 1.32 ppm was assigned to H-17 methyl protons.

Finally, the signals of the remaining three-methylene group protons (H-1, 2, 3) appeared as two broad doublets and three multiplets. The broad doublet at *δ*_H_ 2.39 ppm and the multiplet at *δ*_H_ 1.79–1.74 ppm were, respectively, assigned to H-1*β* and H-1*α* due to the correlations of *δ*_H_ 2.39/1.79–1.74/*δ*_C_ 39.48 ppm in the HSQC spectrum and *δ*_H_ 2.39/H-20, 11, *δ*_H_ 1.79–1.74/H-5, 11 in the ROESY spectrum. Similarly, the broad doublet at *δ*_H_ 1.58 ppm and the multiplet at *δ*_H_ 1.53–1.47 ppm were, respectively, assigned to H-3*β* and H-3*α* based on the correlations of *δ*_H_ 1.58/1.53–1.47/*δ*_C_ 37.52 ppm in the HSQC spectrum and *δ*_H_ 1.58/H-19, *δ*_H_ 1.53–1.47/H-5, 18*α* in the ROESY spectrum. The remaining multiplet at *δ*_H_ 1.85–1.79 ppm was assigned to H-2, which also be demonstrated by the correlations of *δ*_H_ 1.85–1.79/H-3*β*, 3*α*, 1*β*, 1*α* in the COSY spectrum.

#### 2.2.3. ^13^C-NMR Spectrum Analysis of Compound **5**

**Figure 3:**
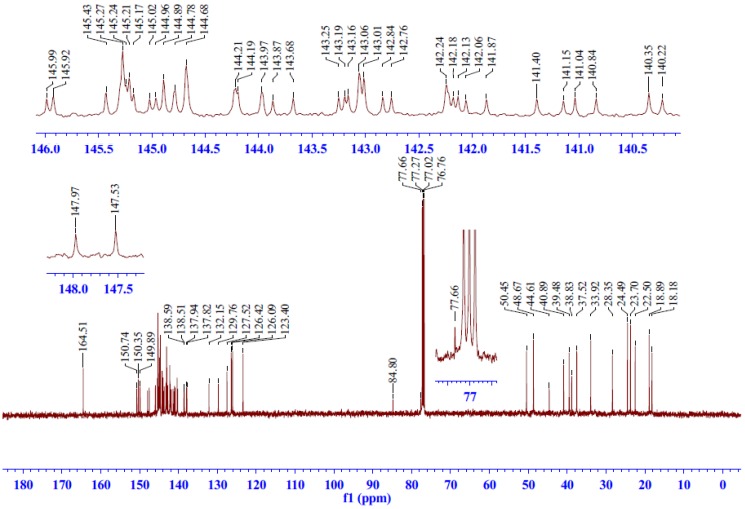
^13^C-NMR spectrum of compound **5**.

The proton noise decoupled ^13^C-NMR spectrum (Figure 3) displayed 24 and 43 resonance signals respectively for (tetrachlorophthaloyl)dehydroabietylamine moiety and C_60_ moiety of compound **5**. In the ^13^C-NMR spectrum, the signal at *δ*_C_ 164.51 ppm could be assigned to C-7', 8', which was supported by the cross peaks of *δ*_C_ 164.51 ppm/H-18*α*, 18*β* in the HMBC spectrum. 

In the HSQC spectrum, the signals at *δ*_C_ 23.70, 24.49, 18.89 and 22.50 ppm were attributed to C-16, 17, 19, 20 methyl carbon atoms due to the cross peaks of *δ*_C_ 23.70/H-16, *δ*_C_ 24.49/H-17, *δ*_C_ 18.89/H-19 and *δ*_C_ 22.50/H-20. In addition, the signals at *δ*_C_ 18.18, 39.48, 37.52, 28.35 and 50.45 ppm were attributed to C-2, 1, 3, 6, 18 methylene carbon atoms due to the cross peaks of *δ*_C _18.18/H-2, *δ*_C_ 39.48/H-1*α*,1*β*, *δ*_C_ 37.52/H-3*α*, 3*β*, *δ*_C_ 28.35/H-6*α*, 6*β* and *δ*_C_ 50.45/H-18*α*, 18*β*. The resonance signals at *δ*_C_ 48.67 and 33.92 ppm were assigned to methine carbon atoms C-5 and C-15 based on the cross peaks of *δ*_C_ 48.67/H-5 and *δ*_C_ 33.92/H-15. The HSQC spectrum also revealed the cross peaks between aromatic hydrogens H-11, 12, 14 and their corresponding carbon atoms. From the HSQC spectrum, we can clearly assign C-11 at *δ*_C_ 123.40, C-12 at *δ*_C_ 126.09 and C-14 at *δ*_C_ 126.42 ppm.

The quaternary carbon atoms were assigned by using the long-range correlated HMBC experiment. The signals at *δ*_C_ 40.89, 132.15, 150.35, 38.83 and 145.43 ppm were assigned to C-4, 8, 9, 10, 13 quaternary carbon atoms because of the long-range cross peaks of *δ*_C_ 40.89 ppm/H-19, 5, 18*α*, 18*β*, 6*β*, *δ*_C_ 132.15 ppm/H-6*β*, 11, *δ*_C_ 150.35 ppm/H-14, 12, 20, 5, *δ*_C_ 38.83 ppm/H-20, 5, 6*α*, 2, 11 and *δ*_C_ 145.43 ppm/H-16, 17, 15, 11. The signals at *δ*_C_ 44.61, 84.80, 77.66 ppm were assigned to C-7 and the sp^3^-hybridized bridgehead carbon atom C-21, 22 on the cyclopropyl moiety due to the long-range cross peaks of *δ*_C_ 44.61 ppm/H-6*α*, 6*β*, 14, 11, *δ*_C_ 84.80 ppm/H-6*α*, 6*β* and *δ*_C_ 77.66 ppm/H-6*α*. This pattern is unambiguously diagnostic for *C_1_* symmetric structure with 6,6-junction [14,15].

Only four signals were observed in the tetrachlorophthaloyl moiety due to the symmetry of C-7', 8', C-1', 6', C-2', 5' and C-3', 4'. The carbon signals at *δ*_C_ 127.52, 129.76, 140.35 ppm can be assigned to C-1', 6', C-2', 5' and C-3', 4' [16]. The remaining 41 resonance signals between *δ*_C_ 150.74–137.82 ppm were attributed to C_60_-sp^2^ carbon atoms (due to the overlapping, only 41 resonance signals for C_60_-sp^2^ carbon atoms were observed, maximum 58 sp^2^-carbon resonance signals).

## 3. Experimental

### 3.1. General

1D (^1^H and ^13^C) and 2D NMR experiments were performed on a Bruker AVANCE AV-500 NMR spectrometer (500.13 MHz for ^1^H and 125.77 MHz for ^13^C). The samples were dissolved in 0.5 mL CDCl_3_, ^1^H-NMR and ^13^C-NMR spectra were recorded using TMS as an internal reference. Chemical shifts were reported in parts per million (ppm). FT infrared (IR) spectrum were recorded as KBr pellets on a Nicolet 360 FT-IR spectrometer and UV-vis spectra on a Shimadzu UV-2550 UV-VIS spectrometer. ESI mass spectrometric were obtained on Agilent 1100 Capillary LC/Micromass Q-Tof Micro mass spectrometer. Matrix-assisted laser desorption ionization time-of-flight (MALDI-TOF) mass spectra was performed on a Bruker Daltonics Autoflex III mass spectrometer using *α*-cyano-4-hydroxycinnamic acid (CHCA) as a matrix in a negative-ion reflector mode. All chemicals and solvents were obtained from commercial sources and used as received or dried according to standard procedures. Column chromatography was performed on silica gel (ZCXⅡ, 100–200 mesh). Chemical reactions were monitored by thin layer chromatography using precoated silica gel GF254 plates.

### 3.2. Preparation of N,N-(Tetrachlorophthaloyl)dehydroabietylamine (***2***)

A mixture of tetrachlorophthalic anhydride (5 g, 17.5 mmol), dehydroabietylamine (5 g, 17.5 mmol), and glacial acetic acid (60 mL) was stirred under N_2_ at 135 °C for 2.5 h. Then the mixture was cooled and poured into 250 mL of ice water. The solid was filtered, washed successively with distilled water and ethanol, dried in vacuo to give the crude product, which was purified by column chromatography on silica gel (petroleum ether-toluene, 2:1) to yield **2** as a white solid (7.11 g, 73.4%), m.p. 220–221 °C. IR *ν*_max_/cm^–1^: 2934, 2860, 1776, 1714, 1630, 1498, 1430, 1392, 1369, 1338, 1297, 1200, 1091, 1069, 879, 820, 736, 556, 476; ^1^H-NMR: *δ* 7.13 (d, *J* = 7.9 Hz, 1H), 6.96 (dd, *J* = 8.2, 1.5 Hz, 1H), 6.91 (s, 1H), 3.68 (d, *J* = 13.7 Hz, 1H), 3.55 (d, *J* = 13.7 Hz, 1H), 2.98–2.96 (m, 2H), 2.81 (septet, *J* = 6.9 Hz, 1H), 2.26 (br d, *J* = 12.5 Hz, 1H), 2.22–2.18 (m, 1H), 1.86–1.79 (m, 1H), 1.75–1.62 (m, 2H), 1.49 (dd, *J* = 13.4, 1.2 Hz, 1H), 1.44–1.28 (m, 3H, H-1*α*), 1.23 (s, 3H), 1.21 (d, *J* = 7.0 Hz, 6H), 1.05 (s, 3H); ^13^C-NMR: *δ* 164.50 (2C=O, imide), 147.09, 145.69, 140.07 (2C, -N(CO)_2_*C*_6_Cl_4_), 134.86, 129.58 (2C, -N(CO)_2_*C*_6_Cl_4_), 127.52 (2C, -N(CO)_2_*C*_6_Cl_4_), 127.05, 123.82, 123.78, 49.89, 45.39, 39.53, 38.09, 37.61, 37.21, 33.44, 30.07, 25.81, 23.96, 23.94, 19.45, 19.06, 18.45; TOF MS (ES^−^) *m/z* 524.3 ([M(^35^Cl_4_)–C_2_H_3_]^−^), 526.2 ([M(^35^Cl_3_^37^Cl)–C_2_H_3_]^−^), 528.2 ([M(^35^Cl_2_^37^Cl_2_)–C_2_H_3_]^−^). Anal. Calcd for C_28_H_29_Cl_4_NO_2_ (553.3): C, 60.78; H, 5.28; N, 2.53. Found: C, 60.37; H, 5.35; N, 2.48.

### 3.3. Preparation of 7-oxo-N,N-(Tetrachlorophthaloyl)dehydroabietylamine (***3***)

To a mixture of CrO_3_ (45 mg, 0.45 mmol) and CH_2_Cl_2_ (90 mL), 65% *t*-BuOOH (11.6 mL, 72.3 mmol) and pyridine (0.073 mL, 0.904 mmol) were added. After stirring at room temperature for 3 min, compound **2** (5 g, 9.035 mmol) was added. Stirring continued at room temperature for 24 h, the mixture was concentrated to about 15 mL *in vacuo* at 30 °C, then poured into methanol (60 mL). The precipitate formed was filtered and washed with methanol to give a crude product, which was purified by column chromatography on silica gel (toluene-ethyl acetate, 20:0.5) to afford compound **3** as a white solid (3.51 g, 68.5%), m.p. 224–226 °C. IR *ν*_max_/cm^–1^: 2961, 2930, 2865, 1777, 1716, 1684, 1608, 1561, 1515, 1489, 1456, 1430, 1377, 1342, 1297, 1248, 1197, 1093, 977, 912, 831, 797, 738, 613, 558, 500, 471; ^1^H-NMR: *δ* 7.89 (d, *J* = 2.1 Hz, 1H), 7.39 (dd, *J* = 8.1, 2.0 Hz, 1H), 7.27 (d, *J* = 7.3 Hz, 1H), 3.64 (d, *J* = 13.7 Hz, 1H), 3.51 (d, *J* = 13.7 Hz, 1H), 3.06 (dd, *J* = 18.0, 3.7 Hz, 1H), 2.93 (septet, *J* = 6.9 Hz, 1H), 2.76 (dd, *J* = 18.0, 14.0 Hz, 1H), 2.32 (br d, *J* = 12.8 Hz, 1H), 2.00 (dd, *J* = 13.9, 3.5 Hz, 1H), 1.75–1.70 (m, 2H), 1.60–1.48 (m, 2H), 1.45–1.34 (m, 1H), 1.28 (s, 3H), 1.25 (d, *J* = 7.0 Hz, 6H), 1.12 (s, 3H); ^13^C-NMR: *δ* 198.61 (C=O), 164.34 (2C=O, imide), 153.18, 146.89, 140.06 (2C, -N(CO)_2_*C*_6_Cl_4_), 132.48, 130.66, 129.60 (2C, -N(CO)_2_*C*_6_Cl_4_), 127.44 (2C, -N(CO)_2_*C*_6_Cl_4_), 125.18, 123.34, 49.71, 45.36, 39.16, 37.86, 37.29, 36.82, 36.34, 33.58, 24.18, 23.81, 18.72, 18.00; TOF MS (ES^+^) *m/z* 565.9 [M(^35^Cl_4_)+H]^+^, 567.9 [M(^35^Cl_3_^37^Cl)+H]^+^, 569.9 [M(^35^Cl_2_^37^Cl_2_)+H]^+^, 571.9 [M(^35^Cl^37^Cl_3_)+H]^+^, 573.9 [M(^37^Cl_4_)+H]^+^; Anal. Calcd for C_28_H_27_Cl_4_NO_3_ (567.3): C, 59.28; H, 4.80; N, 2.47. Found: C, 57.66; H, 4.95; N, 2.31.

### 3.4. Preparation of N,N-(Tetrachlorophthaloyl)dehydroabietylamine p-Tosylhydrazone (***4***)

To a solution of compound **3** (3 g, 5.288 mmol) in benzene (120 mL) and ethanol (30 mL), *p*-toluenesulfonyl hydrazide (1.65 g, 8.86 mmol) and *p*-toluenesulfonic acid (82 mg, 0.476 mmol) were added. The reaction mixture was stirred and refluxed during 8 h under N_2_. After cooling to room temperature, the mixture was concentrated under vacuum, cooled in an ice bath and the precipitate was collected by filtration, and washed with ethanol. The crude product was purified by column chromatography on silica gel (toluene-ethyl acetate, 20:0.4) to yield **4** as a white solid (3.41 g, 87.8%), m.p. 218–220 °C. IR *ν*_max_/cm^–1^: 3224, 3065, 2927, 2860, 1779, 1719, 1595, 1489, 1433, 1380, 1329, 1199, 1163, 1086, 1017, 978, 914, 810, 737, 708, 664, 631, 574, 541, 498, 468; ^1^H-NMR: *δ *8.08 (s, 1H, NH), 8.02 (d, *J* = 8.4 Hz, 2H, *o*-HArSO_2_-), 7.79 (d, *J* = 1.8 Hz, 1H), 7.33 (d, *J* = 8.1 Hz, 2H, *m*-HArSO_2_-), 7.14 (dd, *J* = 8.1, 1.9 Hz, 1H), 7.10 (d, *J* = 8.2 Hz, 1H), 3.61 (d, *J* = 14.1 Hz, 1H), 3.50 (d, *J* = 14.1 Hz, 1H), 3.15 (dd, *J* = 17.3, 4.1 Hz, 1H), 2.87 (septet, *J* = 6.9 Hz, 1H), 2.43 (s, 3H, *CH_3_*ArSO_2_-), 2.40 (dd, *J* = 17.2, 13.4 Hz, 1H), 2.21 (br d, *J* = 12.5 Hz, 1H), 1.73–1.63 (m, 4H), 1.51 (dd, *J* = 13.3, 3.9 Hz, 1H), 1.44–1.35 (m, 1H), 1.24 (d, 3H, *J* = 6.9 Hz), 1.23 (d, 3H, *J* = 6.9 Hz), 1.14 (s, 3H), 1.08 (s, 3H); ^13^C-NMR: *δ *164.46 (2C=O, imide), 153.38 (C=N), 148.89, 146.25, 143.90 (*p*-ArSO_2_-), 140.15 (2C, -N(CO)_2_*C*_6_Cl_4_), 135.69 (*p*-ArCH_3_), 129.77, 129.64 (2C, -N(CO)_2_*C*_6_Cl_4_), 129.42 (2C, *m*-ArSO_2_-), 128.51, 128.42 (2C, *o*-ArSO_2_-), 127.34 (2C, -N(CO)_2_*C*_6_Cl_4_), 123.09, 122.61, 49.36, 42.78, 39.44, 37.28, 37.27, 37.11, 33.53, 23.90, 23.72, 23.63, 21.58 (*CH_3_*ArSO_2_-), 19.03, 18.02; TOF MS (ES^+^) *m/z* 733.9 ([M(^35^Cl_4_)+H]^+^), 735.9 ([M(^35^Cl_3_^37^Cl)+H]^+^), 737.9 ([M(^35^Cl_2_^37^Cl_2_)+H]^+^), 739.9 ([M(^35^Cl^37^Cl_3_)+H]^+^). Anal. Calcd for C_35_H_35_Cl_4_N_3_O_4_S (735.5): C, 57.15; H, 4.80; N, 5.71. Found: C, 57.53; H, 4.71; N, 5.86.

### 3.5. Preparation of 7-C_60_-Adduct of N,N-(Tetrachlorophthaloyl)dehydroabietylamine (***5***)

Compound **4** (408.9 mg, 0.556 mmol) was dissolved in dry pyridine (7 mL) in a dried three-necked flask under N_2_. Then, NaOMe (31.2 mg, 0.578 mmol) was added, and the mixture was stirred for 15 min at room temperature. A solution of C_60_ (200 mg, 0.278 mmol) in chlorobenzene (55 mL) was added and the mixture was stirred at 70 °C for 24 h. After cooling to room temperature the solvent was evaporated *in vacuo*, the residue was column chromatographed on silica gel, pre-eluted with CS_2_ to remove unreacted C_60_ (67.7 mg) and then with CS_2_-CHCl_3_ (10:1) to give 5 as a dark brown solid (145 mg, 41%). IR *ν*_max_/cm^−1^: 3430, 2923, 2858, 1776, 1721, 1628, 1559, 1514, 1459, 1427, 1372, 1334, 1163, 1059, 893, 825, 740, 555, 526, 472; UV-vis (CHCl_3_) λ_max_ (nm): 697.00, 493.00, 436.50; ^1^H-NMR: *δ* 8.40 (d, *J* = 1.8 Hz, 1H), 7.43 (d, *J* = 8.1 Hz, 1H), 7.28 (dd, *J* = 8.1, 1.8 Hz, 1H), 3.88 (d, *J* = 13.6 Hz, 1H), 3.65 (dd, *J* = 14.1, 10.5 Hz, 1H), 3.59 (d, *J* = 13.6 Hz, 1H), 2.99 (septet, *J* = 6.7 Hz, 1H), 2.39 (br d, *J* = 11.9 Hz, 1H), 1.97 (dd, *J* = 10.3, 9.2 Hz, 1H), 1.86 (s, 3H), 1.85–1.79 (m, 2H), 1.79–1.74 (m, 1H), 1.58 (br d, *J* = 13.0 Hz, 1H), 1.53–1.47 (m, 1H), 1.32 (d, *J* = 7.0 Hz, 3H), 1.31 (s, 3H), 1.30 (d, *J* = 6.8 Hz, 3H); ^13^C-NMR: *δ* 164.51 (2C=O, imide), 150.74, 150.35, 149.89, 147.97, 147.53, 145.99, 145.92, 145.43, 145.27, 145.24, 145.21, 145.17, 145.02, 144.96, 144.89, 144.78, 144.68, 144.21, 144.19, 143.97, 143.87, 143.68, 143.25, 143.19, 143.16, 143.06, 143.01, 142.84, 142.76, 142.24, 142.18, 142.13, 142.06, 141.87, 141.40, 141.15, 141.04, 140.84, 140.35 (2C, -N(CO)_2_*C*_6_Cl_4_), 140.22, 138.59, 138.51, 137.94, 137.82, 132.15, 129.76 (2C, -N(CO)_2_*C*_6_Cl_4_), 127.52 (2C, -N(CO)_2_*C*_6_Cl_4_), 126.42, 126.09, 123.40, 84.80, 77.66, 50.45, 48.67, 44.61, 40.89, 39.48, 38.83, 37.52, 33.92, 28.35, 24.49, 23.70, 22.50, 18.89, 18.18; MALDI-TOF MS (matrix: CHCA, reflectron negative) *m/z* 1269.4 M^−^(^35^Cl_4_), 1271.4 M^−^(^35^Cl_3_^37^Cl), 1273.4 M^−^(^35^Cl_2_^37^Cl_2_), 1275.4 M^−^(^35^Cl^37^Cl_3_); Anal. Calcd for C_88_H_27_Cl_4_NO_2_ (1271.97): C, 83.09; H, 2.14; N, 1.10. Found: C, 82.48; H, 2.20; N, 1.04.

## 4. Conclusions

In conclusion, the new compound **5** was synthesized from dehydroabietylamine, the assignments of the proton and carbon signals for 7-C_60_-adduct of *N*,*N*-(tetrachlorophthaloyl)dehydroabietylamine were made possible by using 1D and 2D NMR techniques including ^1^H-, ^13^C-NMR, COSY, ROESY, HSQC and HMBC experiments. The two peaks at *δ*_C_ 84.80, 77.66 ppm in the ^13^C-NMR spectra correspond to the sp^3^-hybridized bridgehead carbons on the cyclopropyl moiety. This pattern is unambiguously diagnostic for *C_1_* symmetric structure with 6,6-junction. All the spectral data support and confirm the proposed structure of the target compound.
